# The Impact of Pinless Navigation in Conventionally Aligned Total Knee Arthroplasty

**DOI:** 10.1155/2018/5042536

**Published:** 2018-02-08

**Authors:** P. Koenen, M. M. Schneider, T. R. Pfeiffer, B. Bouillon, H. Bäthis

**Affiliations:** Department of Orthopaedics, Trauma Surgery and Sports Medicine, Cologne-Merheim Medical Center, University of Witten/Herdecke, Cologne, Germany

## Abstract

**Background:**

Restoration of the mechanical axis is a main objective in total knee replacement (TKR). Aim of this study was to analyse the verification tool of a pinless navigation system in conventional TKR (cTKR).

**Methods:**

In a prospective study, 147 TKR were performed by conventional technique. Using the “pinless verification” mode of a smartphone based navigation system, the cutting block position and final resection plane for distal femur and proximal tibial resection were measured. If necessary, the block position or resection level were optimized, corrections were protocolled. Postoperatively, standardized radiographs were performed.

**Results:**

In 65.3%, intraoperative measurements changed the surgical procedure (corrections: 20.4% femoral, 25.9% tibial, 19% both). The additional time for surgery compared to cTKR averaged 6 minutes (79 ± 15 versus 73 ± 17 minutes). Using navigation data, the final femoral and tibial axes were in 93% within a range of ±2°. A mean difference of 1.4° and 1.6° could be shown between the final measurement of the navigation system and the postoperative mLDFA and mMPTA.

**Conclusion:**

Intraoperative pinless navigation has impact on the surgical procedure in the majority of cTKR. It represents a less time-consuming tool to improve implant position while maintaining the routine of conventional technique.

## 1. Introduction

Total knee replacement (TKR) has been established as standard therapy for severe osteoarthritis. Restoration of the mechanical axis is a main objective in TKR, as it is attributed to good long-term results. Numerous radiological and clinical studies have proven that computer-assisted total knee replacements (CAS-TKR) are more precise regarding limb alignment reconstruction as well as implant position compared to the conventional technique [1–3]. Moreover registry data from the Australian joint registry have demonstrated a reduced revision rate of CAS-TKR compared to conventional technique for younger patients on a large patient data base [4]. In spite of its valuable advantages, the navigation technique is still not used as routine [5]. Main limitations are higher costs and additional time required for the surgical procedure [6, 7]. Further disadvantages are a prolonged training curve for new users [3, 8] and morbidity due to the placement of bony reference arrays such as fractures and infections [9, 10]. However, in conventional TKR, no standardized intraoperative technique is available to verify the result of surgery with respect to limb alignment. Only navigation technology offers the opportunity to improve the accuracy of the procedure. In order to bridge this gap and to address some of the aforementioned disadvantages of the navigation technique, recent developments have focused on the introduction of more user-friendly devices and workflows. Pinless navigation systems have been developed to offer an intraoperative verification tool for conventional arranged cutting guides without the need of reference arrays fixed to the bone of the patient [11].

The aim of the study was to analyse the verification tool of a pinless navigation system in conventional total knee arthroplasty. Furthermore, it was questioned whether verification data of a pinless navigation system had impact on the surgical procedure and therefore may affect alignment in conventional total knee arthroplasty. It was hypothesized that in the majority of conventional TKR intraoperative acquired pinless navigation data influence the surgical procedure as defined by a correction of the cutting block position or resection plane.

## 2. Materials and Methods

### 2.1. Study Design

In a prospective study, 147 consecutive TKR were included. Approval was given by the local ethics committee (113/2012). For all knee arthroplasties a cemented, cruciate-retaining implant design was used (PFC Sigma Total Knee System, DePuySynthes Orthopedics, Kirkel, Germany). Surgery was performed by one senior surgeon. The “pinless verification” module of the image-free, smartphone based navigation system DASH (BrainLAB, Munich, Germany) was used, which is also implemented within the “Knee3” software-module of the company.

Exclusion criteria were age < 18 years, the absence of a written consent, and the use of a semiconstrained or constrained implant design.

### 2.2. Navigation System

The DASH system works as an image-free navigation system. All joint information is digitized during surgery without the need for additional preoperative diagnostic. Within the “pinless verification” workflow no reference arrays have to be attached to the femoral or tibial bone. The essential hardware is provided by a sterile draped Apple iPod touch® that is included into a handheld cradle and serves as the operating and display unit. The iPod works remotely with the separated computer platform that is included into the infrared-camera stand using secured Wireless-LAN connection. The software can be used, as preferred by the surgeon, femur or tibia first.

### 2.3. Surgical Technique

All arthroplasties were performed in tibia first technique. The conventional alignment technique was applied (DePuy HP-Instruments-Set®) using extramedullary alignment at the proximal tibia and intramedullary alignment at the distal femur. The femoral alignment guide was set to 5–7° valgus dependent on the preoperatively determined anatomical-mechanical axis angle (AMA-angle). Within the “pinless verification” workflow of the DASH system the resection guide is placed to a preliminary resection position using the conventional instruments. Without any additional arrangement a limited number of anatomical landmarks (femur: Whiteside line, tibia: insertion of the anterior cruciate ligament, medial and lateral malleolus) have to be digitized using the handheld cradle to acquire an accurate 3D position of the cutting block. The surgeon gets instant and comprehensive information of the resection level, the flexion/extension position, and the varus/valgus alignment, which is displayed on the sterile draped iPod. If the position was satisfying the surgery proceeded. If a relevant displacement of the cutting jig of at least 1.5° varus/valgus was measured, correction was performed and the result was digitized again. A cut-off value of 1.5° varus/valgus was chosen, as a range of ±2° is considered as safe zone for individual axes of tibia and femur. A tibial slope of 5° was aimed; correction was performed if this value was missed by at least 2°. Verification of rotational orientation of the femoral component is not supported by the system and was determined ligament-balanced using conventional spacer blocks. For verification of the performed resection plane, a maximum of two landmarks (femur: centre of the femoral head by hip pivoting, tibia: medial and lateral malleolus) have to be digitized. Again, only in case of satisfying alignment data the surgery proceeded; otherwise correction was performed and again the result was measured.

**Figure 1:**
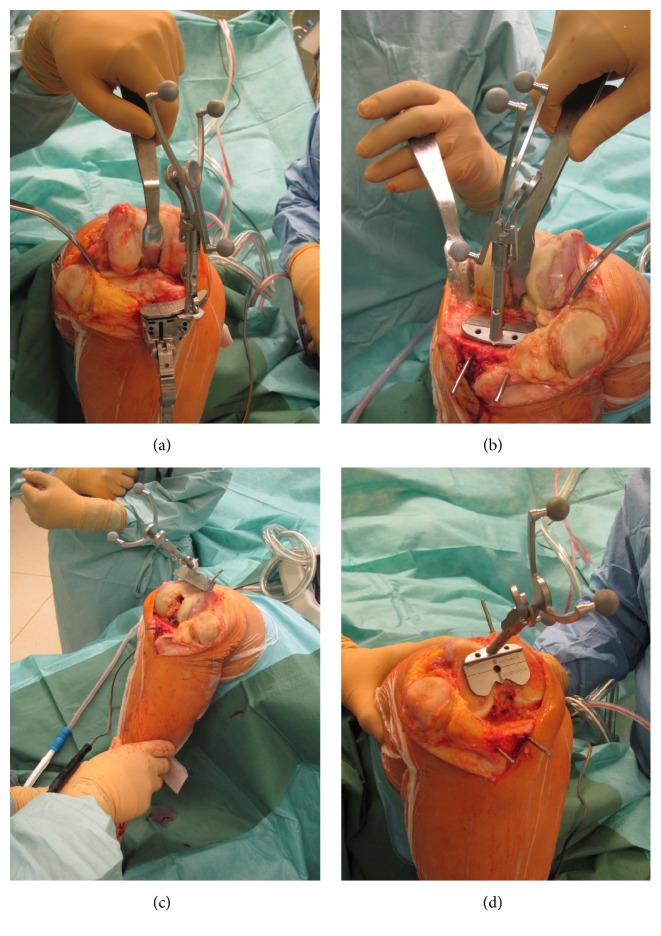
Surgical procedure using the “pinless verification” workflow of the DASH system. (a) Cutting block verification at the proximal tibia, (b) cut verification at the proximal tibia, (c) cutting block verification at the distal femur, and (d) cut verification at the distal femur.

These data were stored for the final patient report. It was recorded whether and to which degree the alignment data of the navigation system were used to optimize the cutting block position or the final resection plane by the surgeon. The surgical procedure is illustrated in Figure 1.

### 2.4. Operating Room Time

Time needed for the navigation process including all coronal bone resections and measurements of the navigation system was measured. Furthermore, the length of the surgical procedure (skin to skin) was documented for each patient. OR (operating room) time was compared to a control group including 125 conventional TKR performed in 2012 by the same senior surgeon.

### 2.5. Full-Length Weight-Bearing Radiographs

Axial limb alignment was evaluated on standardized full-length weight-bearing radiographs before and after the surgery. Radiographs were performed according to an internal standardized protocol based on the recommendations described by Cooke et al. [12]. Postoperative radiographs were delayed until full knee extension was achieved to minimize errors due to incorrect rotation or knee flexion. Alignment measurements were performed using the digital planning software mediCAD version 2.20 (Hectec, Niederviehbach, Germany).

### 2.6. Statistical Analysis

Statistical analysis was performed using GraphPad Prism 6 (La Jolla, California, USA). Means, standard deviations, and ranges were calculated. An unpaired, two-tailed *t*-test was performed to compare OR times of cTKR and DASH TKR. A paired, two-tailed *t*-test was performed to compare differences between the initial conventional alignment and the final resection plane as well as differences of the final measurement and postoperative mLDFA/mMPTA. Values of *p* ≤ 0.05 were considered statistically significant.

## 3. Results

147 computer-assisted primary total knee replacements were included. 48 patients (32.7%) were male and 99 (67.3%) were female. Their mean age was 65 years ranging from 27 to 87 years. 73 (49.7%) patients had surgery on the right knee; 74 (50.3%) on the left knee. The preoperative mechanical axis measured by X-ray was between 24.5° varus and 22.9° valgus, respectively.

**Figure 2:**
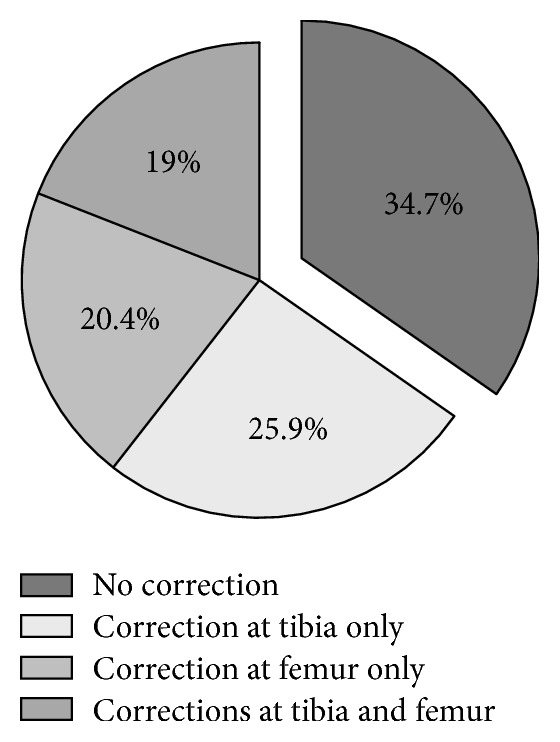
Change of surgical procedure due to the intraoperatively acquired data of the navigation system, presented as percentage of all arthroplasties.

**Figure 3:**
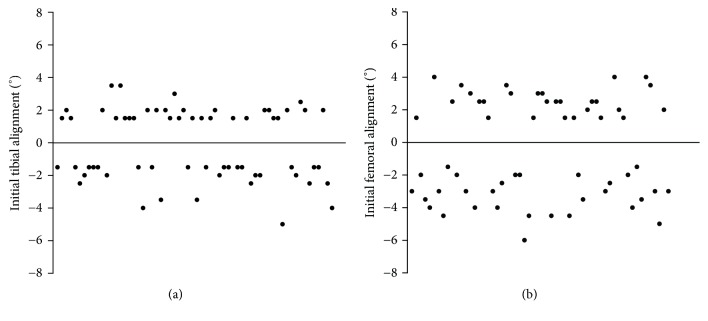
Initial tibial (a) and femoral (b) alignment by using the conventional extramedullary (tibia) and intramedullary (femur) alignment technique. Only cases, in which corrections were performed (− varus, + valgus), are shown.

**Figure 4:**
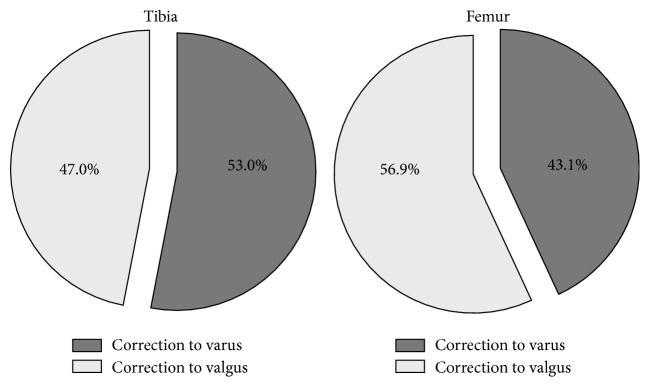
Change of surgical procedure, presented as percentage for the femoral and tibial bone resection, separately. The direction of the required correction is shown.

In 63.3% (93), the intraoperative measurements of the navigation system had impact on the surgical procedure. In 20.4% (30) only the femoral resection and in 25.9% (38) only the tibial resection were modified. In 19.0% (28) of TKR both the femoral and tibial resection were modified within the same surgical procedure while in 34.7% (51) the initial conventional cutting block position was maintained (Figure 2). The initial orientation of the tibial cutting block ranged from 5° varus to 3.5° valgus. The initial orientation of the femoral cutting block was between 6° varus and 4° valgus, demonstrating a substantial variability with a significant number of outliers of the conventional technique (Figure 3). Moreover, in 11.8% (18) both the initial femoral and tibial orientation of the cutting block were displaced to the same direction. Readjustment of the cutting block in the coronal plane or a correction of the final resection plane was carried out in equal parts in the direction of varus and valgus, respectively (femur 43.1% and 56.9%, tibia 53% and 47%; Figure 4). The mean degree of correction was 1.5 ± 1.4° (0–7.5°) at the femur and 1.2 ± 1.0° (0–6.5°) at the tibia. Differences between the initial conventional alignment and the final resection plane were shown to be statistically significant for femur (*p* = 0.02), but not for tibia (*p* = 0.29).

**Figure 5:**
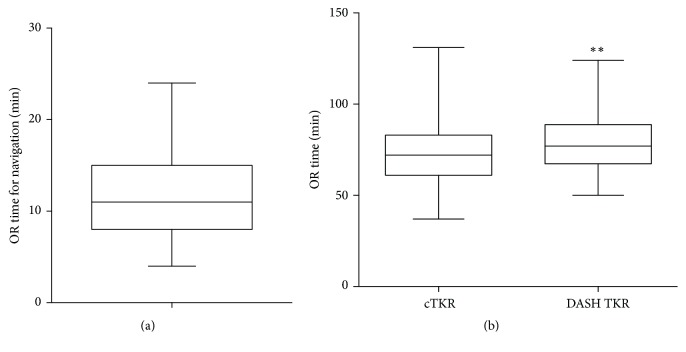
(a) Expenditure of time for all coronal bone resections and measurements of the navigation system, presented as boxplot. (b) OR time for DASH TKR (*n* = 144) and conventional TKR (cTKR; *n* = 125), shown as mean ± SD. ^*∗∗*^*p* < 0.01.

The mean duration of surgery was 79 ± 15 minutes in the navigation group compared to 73 ± 17 minutes in the conventional group, which was statistically significant (*p* = 0.005). The overall time for all coronal bone resections and measurements of the navigation system averaged 11.5 ± 4.5 minutes (Figure 5).

By navigation data the final femoral and tibial axis were in 93% within a range of ±2°. A mean difference of 1.4 ± 1.2° (0–5.4°) and 1.6 ± 1.2° (0–5.0°) could be shown between the final measurement of the navigation system and the mLDFA (mechanical Lateral Distal Femoral Angle) and mMPTA (mechanical Medial Proximal Tibial Angle) in the postoperative full-length weight-bearing radiograph, respectively. Differences were not statistically significant (mLDFA: *p* = 0.39, mMPTA: *p* = 0.55).

## 4. Discussion

Restoration of the mechanical axis is one of the main objectives in TKR. Earlier studies have shown that an alignment in the coronal plane within the range of 3° varus/valgus is associated with a better survival of the prosthesis [1, 2, 8, 13, 14]. Considering various meta analyses from different groups, CAS in total knee arthroplasty has been proven to be more accurate regarding restoration of the mechanical axis compared to the conventional technique [1–3]. Despite this valuable advantage, CAS-TKR has not yet become routine due to disadvantages like additional costs and prolonged procedure time [6, 7]. However, besides navigation technology, no standardized technique is available to verify the implant position and leg alignment in TKR intraoperatively. This is in strict contrast to trauma surgery, where intraoperative X-ray technology is used as a matter of routine to verify the reposition of a fracture or implant position, when performing osteosynthesis.

This study shows that only in one-third of arthroplasties the initial conventional alignment was considered satisfying by the surgeon, accepting a maximum displacement of the resection plane of 1.5° or less from the neutral alignment position. In our study, the initial femoral and tibial alignment by the conventional technique showed a significant number of outliers. Moreover, in 11.8% of cases both the initial femoral and tibial alignment deviated in the same direction. As a consequence maintaining the initial alignment would have led to a substantial malalignment. Therefore, in the majority of arthroplasties the intraoperatively acquired data of the navigation system did have a significant influence on the surgical procedure. This is in accordance with various comparative studies, describing that implant alignment and a resulting leg alignment within a range of ±3° are only achieved at most 80% using the conventional technique [2, 13, 15]. In addition there is no learning curve in improving leg alignment with improved surgical experience [16].

By now, only navigation technology is able to improve the accuracy of the procedure. The described verification module of the DASH system using pinless technique offers the opportunity to bridge this gap. It may be used as intraoperative verification tool in conventional TKR to monitor implant position and to correct the resulting alignment during the surgical procedure. Pinless navigation systems have been developed to address some of the drawbacks of the navigation technique. While lacking disadvantages as the morbidity of Steinman pins or lengthening of OR time [11], pinless navigation systems have been shown to be comparable in accuracy to conventional computer-assisted surgery. The concept of the DASH system, presenting the information in line with the working field of the surgeon, leads to an instant visual feedback of the surgeon's movements within the surgical field. The simplified software algorithm and the intuitive handling have been shown to facilitate the computer-assisted surgical procedure even for navigation beginners [17]. Furthermore, this new technique represents a great possibility for surgical trainees in the context of learning the conventional technique of TKR [18]. However, the DASH system does not provide all features of established navigation systems. Verification of rotational alignment of the femoral component is not supported by the system, as well as a ligament balancing support. An additional option for defining the rotational orientation of the femoral component is not implemented to the pinless verification workflow as freehand navigation techniques have not been able to demonstrate superior precision of rotational implant orientation due to the difficult digitization of landmarks for short axes, for example, epicondylar axis [19–21]. Therefore the rotational alignment within this study was performed in a conventional ligament-balanced technique using spacer blocks.

The precision of the DASH system could be shown to be comparable to established navigation systems with regard to reconstruction of the limb alignment. The final tibial and femoral axes were in 93% within a range of ±2°, which is considered as safe zone for individual axes of tibia and femur.

We measured a mean expenditure of time for the whole navigation process of 11.5 minutes. The surgical procedure was extended by only 6 minutes compared to the conventional technique. In contrast, Bauwens et al. found that the use of established navigation systems extended OR time by 15 minutes [7].

Limitations of the study are mainly due to a lacking control group. In spite of a prospective design we did not include a control group. Therefore, conclusions regarding radiological outcomes are restricted.

## 5. Conclusion

Pinless navigation using a verification workflow as an augmentation to the conventional technique of TKR is a helpful tool to verify cutting block position and resection plane while using the conventional alignment technique. Thereby an improved implant position might be achieved, whereas only a short additional OR time is required. Major disadvantages associated with established navigation systems like fixation of reference arrays or severely increased OR time are eliminated with this technique.
